# 2731. Posa-bly the Better Option? Comparison of Posaconazole vs. Itraconazole for Antifungal Prophylaxis After Heart Transplantation

**DOI:** 10.1093/ofid/ofad500.2342

**Published:** 2023-11-27

**Authors:** Darra Drucker, Ralph Tayyar, Aruna Subramanian, Roy Lee, Jeffrey Teuteberg, Kiran Khush, Helen Luikart, William Alegria, Erik Henricksen

**Affiliations:** University of Washington Medicine, Irvine, California; Lankenau Medical Center, Wynnewood, PA; Stanford University, Palo Alto, California; Stanford Health Care, Stanford, CA; Stanford Medicine, Stanford, California; Stanford University, Palo Alto, California; Stanford Medicine, Stanford, California; Stanford Health Care, Stanford University School of Medicine, Stanford, California; Stanford Medicine, Stanford, California

## Abstract

**Background:**

The American Society of Transplantation recommends targeted Aspergillus prophylaxis after heart transplant (HT). Itraconazole (itra) has historically been used as it is less broad in activity and cheaper than posaconazole (posa). Given the limited data comparing the two, the purpose of this study was to compare the safety and efficacy of posa IV/delayed release tablets vs. itra oral suspension in adult HT recipients.

**Methods:**

Single-center retrospective analysis of HT recipients from January 2015 to December 2021. Both cohorts received inhaled amphotericin b as adjunctive therapy during their index hospitalization. Patients were excluded if they were heart-lung or heart-liver transplants, transitioned to another institution, or expired within 7 days of HT. Fungal infection was defined based on the 2020 consensus definitions of invasive fungal disease by The European Organization for Research and Treatment of Cancer and the Mycoses Study Group Education and Research Consortium. Toxicity of agents was defined as elevations in liver transaminases > 200 IU/mL.

**Results:**

A total of 240 HT were included, 137 with itra and 103 with posa. Patients receiving itra were more likely to have been induced with antithymocyte globulin than patients receiving posa (89% vs 60%, p< 0.001), otherwise baseline characteristics were similar. A total of 8 (5.8%) fungal infections were observed in the itra cohort and 0 (0%) infections for posa (**Table 1**). Patients receiving itra were more likely to develop an infection in the first year (**Figure 1**) but had similar overall survival rates (**Figure 2**). Incidence of elevations in alanine aminotransferase > 200 IU/mL (p=1.0) or in aspartate aminotransferase > 200 IU/mL (p=0.135) was similar between cohorts 2 weeks after antifungal initiation. The most common reason for itra discontinuation was subtherapeutic/undetectable drug levels, and for posa it was most associated with the need to switch to another agent due to drug availability and/or access issues (**Figure 3**) There was no observed difference in the percent of patients who needed to switch to another agent due to adverse effects (p >0.05).

**Figure:**
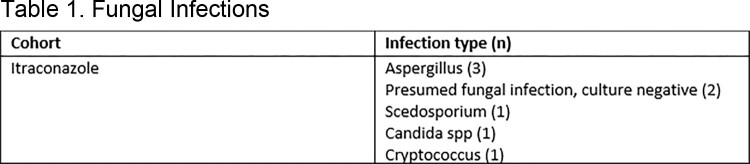

Fungal infections that developed in patients that received itraconazole for antifungal prophylaxis.

**Figure:**
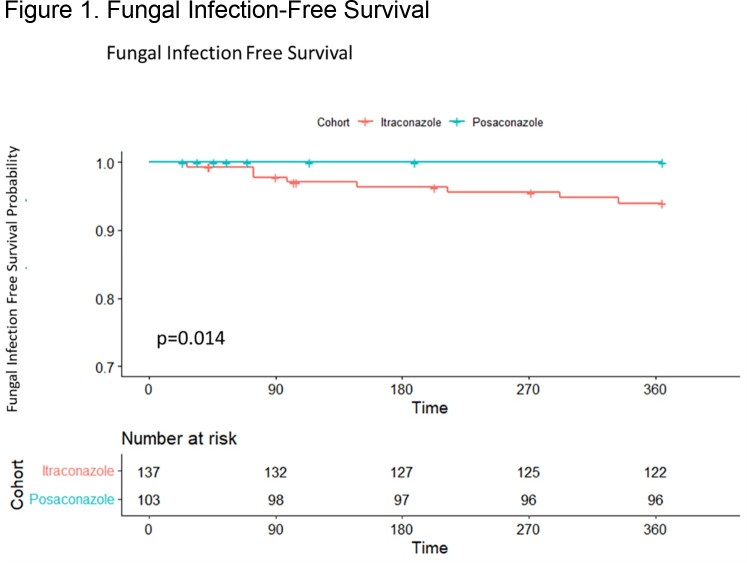

Patients receiving itraconazole for antifungal prophylaxis were more likely to develop an infection in the first year after transplant compared to patients receiving posaconazole.

**Figure:**
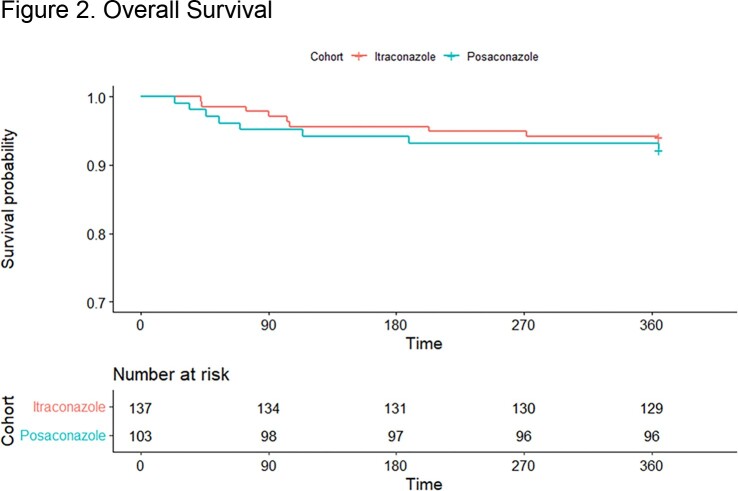

Patients receiving itraconazole and posaconazole for antifungal prophylaxis had similar overall survival rates in the first year after transplant.

**Conclusion:**

Itra and posa had similar safety and tolerability in HT recipients. However, itra was associated with lower fungal infection-free survival in the first year after HT.

**Figure:**
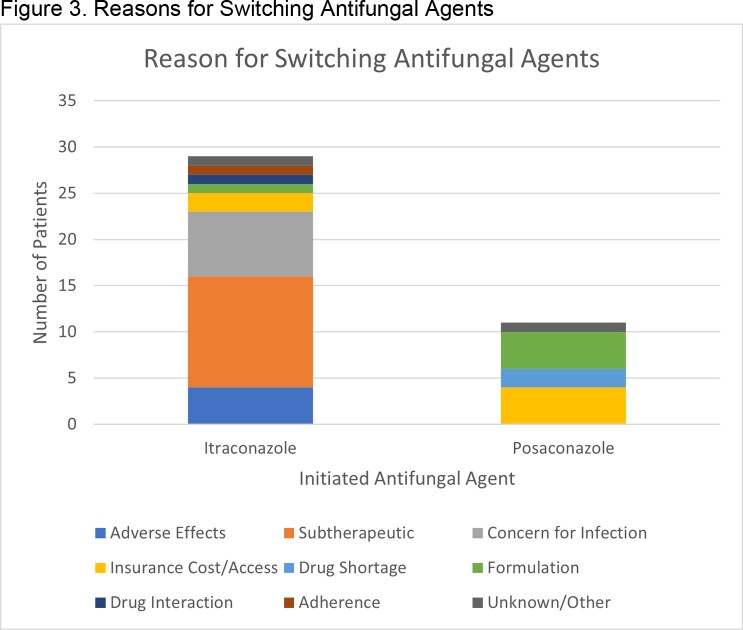

Reasons why patients initiated on itraconazole and posaconazole for antifungal prophylaxis needed to switch to another antifungal agent.

**Disclosures:**

**All Authors**: No reported disclosures

